# Sustained Drug Release From Liposomes for the Remodeling of Systemic Immune Homeostasis and the Tumor Microenvironment

**DOI:** 10.3389/fimmu.2022.829391

**Published:** 2022-04-12

**Authors:** Anjie Zheng, Fang Xie, Sanyuan Shi, Shounan Liu, Jinfeng Long, Yuhong Xu

**Affiliations:** ^1^ School of Pharmacy, Shanghai Jiao Tong University, Shanghai, China; ^2^ Yunnan Key Laboratory of Screening and Research on Anti-pathogen Plant Resources in Western Yunnan, Dali University, Dali, China

**Keywords:** immune homeostasis, myeloid - derived suppressor cell, all-trans rethoric acid (ATRA), liposome, active loading method, dentric cell

## Abstract

Myeloid Derived Suppressor Cells (MDSCs) play important roles in constituting the immune suppressive environment promoting cancer development and progression. They are consisted of a heterogeneous population of immature myeloid cells including polymorphonuclear MDSC (PMN-MDSC) and monocytes MDSC (M-MDSC) that are found in both the systemic circulation and in the tumor microenvironment (TME). While previous studies had shown that all-trans retinoic acid (ATRA) could induce MDSC differentiation and maturation, the very poor solubility and fast metabolism of the drug limited its applications as an immune-modulator for cancer immunotherapy. We aimed in this study to develop a drug encapsulated liposome formulation L-ATRA with sustained release properties and examined the immuno-modulation effects. We showed that the actively loaded L-ATRA achieved stable encapsulation and enabled controlled drug release and accumulation in the tumor tissues. *In vivo* administration of L-ATRA promoted the remodeling of the systemic immune homeostasis as well as the tumor microenvironment. They were found to promote MDSCs maturation into DCs and facilitate immune responses against cancer cells. When used as a single agent treatment, L-ATRA deterred tumor growth, but only in immune-competent mice. In mice with impaired immune functions, L-ATRA at the same dose was not effective. When combined with checkpoint inhibitory agents, L-ATRA resulted in greater anti-cancer activities. Thus, L-ATRA may present a new IO strategy targeting the MDSCs that needs be further explored for improving the immunotherapy efficacy in cancer.

**Graphical Abstract:**
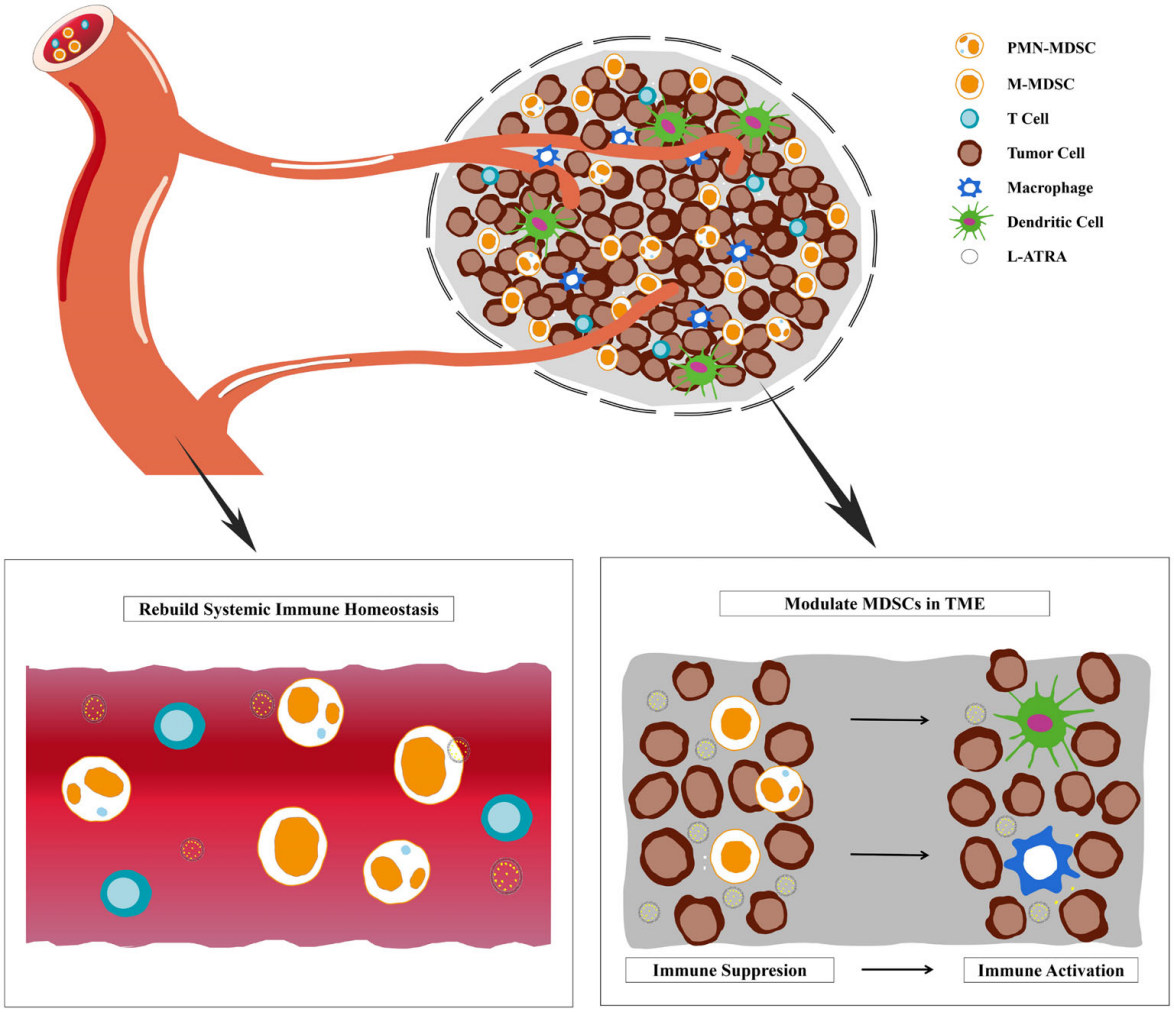
Liposome sustainable release of ATRA enable remodeling immune homeostasis and improving CTL activities inside tumor microenvironment.

## Introduction

The detrimental roles of immature myeloid cells in immune-oncology have been discussed extensively in many studies (1, 2). These heterogeneous population of cells with impaired immune stimulation functions are named myeloid-derived suppressor cells (MDSCs). Higher numbers of MDSCs were found in cancer patients and their accumulation in the tumor tissues constituted the immune suppressive tumor microenvironment (TME) (1, 2). In patients with Glioblastoma, the numbers of circulating M-MDSCs were higher than those in patients with benign and grade I/II glioma (3). Greater numbers of MDSCs in circulation and tumor infiltrates suggested poorer prognosis. Studies had indicated that TME MDSCs contributed to chemoresistance, as well as resistance to immune checkpoint inhibitors (4–6). They were also found to correlate with the treatment outcomes of Ipilimumab in melanoma patients (7).

There have been various therapeutic strategies proposed for targeting MDSCs for therapeutic benefits, including MDSC depletion and/or deactivating (8). The effects of all-trans retinoic acid (ATRA) on immature myeloid cells (a.k.a. MDSCs) were first reported by Gabrilovich and coworker and later examined in many studies for cancer immunotherapy (9, 10). Kusmartsev et al. described the transformation of immature myeloid cells into mature DC, macrophages, and granulocytes after ATRA treatment in mouse tumor models (11). Tobin et al. reported the use of ATRA in combination with Ipilimumab to treat stage IV melanoma patients (12). Their data all suggested that ATRA could significantly improve CD8^+^ T cells activation and decrease the frequency of circulating MDSCs. Therefore, ATRA was considered as a potent immuno-modulator with valuable potentials in immune-oncology.

Nevertheless, ATRA is very poorly soluble and subjects to fast metabolism by endogenous vitamin A clearance pathways (13, 14). Thus its systemic exposure as well as pharmacodynamic effects on MDSCs were limited even with oral doses up to 150 mg/m^2^/day (9). The drug concentration in solid tumor tissues should be even lower considering the very short T_1/2_ of about 1 hour. In order to improve its bioavailability to reach effective exposure, several groups had attempted various nano drug delivery approaches (15–18). Aronex Pharmaceuticals developed an ATRA liposomal product (ATRAGEN™) and enabled i.v. dosing of up to 150mg/m^2^ (19). Similarly, ATRA was co-formulated in suspensions and dosed with various drug combinations (15, 17, 18, 20, 21). Mirza et al. examined the PK data after oral dosing of ATRA in their clinical studies and found that the MDSC PD effects were only seen in patients with plasma concentration higher than 150 ng/ml (9). Therefore, we hypothesized that it is of critical importance to maintain the plasma concentration and also increase the intratumor drug distribution for both systemic and local modulation effects. In this study, we made liposome formulations containing actively loaded ATRA (L-ATRA) with passive targeting capacities towards MDSCs. Since MDSCs are found either patrolling the circulation system or recruited inside TME, the effect of L-ATRA should have two folds. One in remodeling the immune homeostasis and the other improving CTL activities inside TME. Both aspects need to be evaluated in order to understand the immune modulation effects of L-ATRA and its potential in cancer immunotherapy.

## Materials and Methods

### Materials

Hydrogenated soybean phosphatidylcholine (HSPC), 1, 2-distearoyl-sn-glycero -3-phosphoethanolamine-N-[methoxy(polyethyleneglycol)-2000] (DSPE-PEG2000) were purchased from Lipoid (Germany). Cholesterol and fluorescein labeled PEG-DSPE were from Avanti Polar Lipids (AL, USA). All-trans retinoic acid (ATRA) was obtained from Shandong Liangfu Pharmaceutical Co., Ltd. Other reagents including ethanol, calcium acetate, sodium chloride, potassium chloride and sucrose were of analytical reagent grades and purchased from Sinopharm (Shanghai, China). Fetal bovine serum and hydroxypropyl-β-cyclodextrin (HPCD) were from Sigma-Aldrich (Steinheim, Germany). Acetonitrile and methanol (HPLC grade) were from Thermo Fisher Scientific (Waltham, USA). Therapeutic mouse anti-PD-1 were purchased from BIO X CELL™(USA), InVivoMAb Antibodies,anti-mouse PD-1(CD279,Clone: 29F.1A12).

### ATRA Quantification Using HPLC and UPLC/MS-MS

ATRA was dissolved in methanol for HPLC quantification. The mobile phase consisted of acetonitrile, 2% acetic acid solution and methanol (57.5: 25:17.5, *v*:*v*:*v*). Injection volume was 30 μL. The velocity of mobile phase was 1 mL/min and the detection wave length was 340 nm.

Plasma and tissue samples containing ATRA were quantified using a triple quadrupole 5500 UPLC/MS-MS (AB Sciex, USA). Briefly, 150 µL ACN containing the internal standard (Acitretin, 50 ng·mL^-1^) was added into 30 µL of samples, vortexed for 10min, and centrifuged at 10000 rpm for 10min. 70 µL supernatant was collected, diluted with 70 µL of dd H_2_O, and vortexed for 10min. An aliquot of 15 µL of the mixture was injected into the LC-MS/MS system. The elution phase consisted of 85% water and 15% acetonitrile (0.1% NH_3_·H_2_O), changed to 15% water and 85% acetonitrile (0.1% NH3·H_2_O) in 1.6 min, followed by 1.0 min elution at 15% water, then a jump to 85% water and 15% acetonitrile (0.1% NH_3_·H_2_O) within 0.01 min, and finally 1.39 min with 85% water and 15% acetonitrile (0.1% NH_3_·H_2_O). The velocity of the mobile phase was 0.4 mL/min. The signals at *m/z* 299.4→ 255.2 for ATRA and *m/z* 326.9→177.1 for Acitretin were collected in negative ion mode with an electrospray ionization source.

### ATRA Liposomes Preparation

ATRA lipid co-suspensions were made by dissolving ATRA, HSPC, cholesterol, and DSPE-PEG200 in ethanol and injecting into buffers containing 10 mM HEPEs. Then the mixture was extruded through a polycarbonate membrane (Nucleopore Co., Canada) using the Northern Lipid extruder.

For actively loaded L-ATRA, empty liposomes were prepared by ethanol injection in calcium acetate buffers and extruded through a polycarbonate membrane. They were dialyzed in 10mM HEPEs buffers (pH 7.0), mixed with ATRA-HPCD (1:3 mol ratio) solution in HEPEs buffer pH 7.5, and incubated at 60°C for 40 min.

Both liposome samples were dialyzed at least 3 times against 1000x volume of 10mM HEPEs buffer (pH 7.0) with a 100 kDa cut-off membrane (Millipore, USA) to remove unencapsulated ATRA. The encapsulation efficiency was calculated as the percent of drug remaining over total added drugs. The size distribution and polydispersity index (PDI) were determined using Zetasizer 3000HSA (Malvern Instruments, UK).

The L-ATRA was analyzed using the MicroCal-VP differential scanning calorimeter (DSC) (GE Instrument Corporation, Sweden). Cyro-EM images were obtained using a Talos F200C electron microscope (FEI, USA).

The *in vitro* drug release studies were conducted by adding 1ml of liposomes in dialysis tubes with a 100 kDa cut-off membrane. The dialysis tubes were place in 1L of PBS containing 20% of BSA and incubated at 37°C with constant stirring. 20µL aliquots were taken at various time points and quantified.

### Cell Isolation and Culture Procedures

The murine colorectal carcinoma cell line CT26, human lung cancer cell line A549, and human leukemia cell lines HL-60 and NB4 were obtained from The Cell Bank of Type Culture Collection of Chinese Academy of Sciences. They were cultured in RPMI 1640 medium (GIBCO, USA) containing 10% FBS (PAA Laboratories, AU), 100 U/mL streptomycins and 100 U/mL penicillin (Invitrogen, USA).

PBMCs from mouse CT26 tumor models and cancer patient blood samples were isolated using regular procedures. Briefly, blood samples were drawn into sodium heparin anticoagulant collection tubes and diluted with phosphate-buffered saline (PBS). Red blood cells were depleted using Red Blood Cell Lysis Buffer (BioLegend, USA). PBMCs were collected after centrifugation at 800g for 15 min at room temperature using Ficoll-Paque™ PLUS (GE Healthcare, Sweden) and washed with PBS.

STEMCELL EasySep™ CD11b Positive Selection Kit II was used to isolate myeloid cells in tumor tissue. Briefly, tumor tissues were harvest and minced into a homogenous paste (< 1 mm pieces) using a razor blade or scalpel. Then they were digested with Dispase II(1.2 U/ml) in RPMI 1640 at 37°C for 30 minutes. After digestion, cells were collected and resuspended in EasySep™ buffer, and CD11b+Cells were isolated using the EasySep™ Magnet. Cell population purity was confirmed by Flow cytometry and exceeded 90%.

### FACS Analysis

For flow cytometry analysis, cells were washed twice in DPBS depleted of magnesium or calcium, incubated with Aqua Live/Dead stain (Thermo Fisher Scientific) for 20 minutes, and washed again in DPBS supplemented with 2% FBS. The cells were chilled for 20 minutes on ice, washed twice, and then stained with antibodies for various myeloid cell surface antigens as listed in **Supplementary Material**. After staining, the cells were washed again twice in DPBS with 2% FBS and loaded onto a BD LSRFortessa™ X-20 (BD Biosciences) for analysis. Human MDSCs were defined as HLA-DR^lo/-^ CD33^+^ cells. Mouse M-MDSCs were identified as CD45^+^CD11b^+^Ly6C^+^Ly6G^-^ cells and PMN-MDSCs as CD45^+^CD11b^+^Ly6C^+^Ly6G^+^ cells. The respective gating parameters were shown in **Supplemental Figure 3**.

The FACS data were further processed using the t-distributed stochastic neighbor embedding (tSNE) algorithm with 1000 iterations and a perplexity parameter of 50. The graphic presentation with reduced dimensionality was peudo-colored into cell clusters based on the specific marker expression profiles.

### Animal PK and Efficacy Studies

The mouse study protocols were approved by Shanghai Jiao Tong University Animal Care and Use Committee. Balb/C mice and nude mice were obtained from the Shanghai SLAC Co. Ltd. They were housed with free access to food and water until they reach the weight of 20–22 g. Tumor cells were propagated *in vitro* for two passages prior to implantation and injected cells were greater than 90% viable. 1 × 10^6^ CT26 tumor cells or 1 × 10^6^ A549 tumor cells were implanted subcutaneously (s.c.) into shaved flanks of recipient Balb/c mice or nude mice.

For the pharmacokinetics and tissue distribution studies in mice, L-ATRA was injected intravenously at a drug dose of 10 mpK. A total number of 40 mice were included in each group. At specific time points, blood samples were drawn and processed for ATRA quantification. Serum RBP4 levels were measured using the mouse RBP4 ELISA Test Kit (Abcam, USA). Similarly, tissue samples including liver, spleen, kidneys, heart, and tumor were collected at specified time points and analyzed.

The Pharmacokinetic studies in beagle dogs were carried out by 3D BioOptima (Suzhou, China) under GLP conditions. The L-ATRA was labeled with trace amount of deuterated cholesterol (Cambridge Isotope Laboratories Inc, MA), and dosed at 5mg/kg dose. Blood samples were drawn at specific time points, and plasma ATRA, oxo-ATRA, and deuterated cholesterol concentrations were determined using HPLC-MS.

Both the CT26 and A549 tumor bearing mice were treated with intravenous injections of PBS (vehicle) or L-ATRA (10 mg/kg). The injections were given at every other day for a total of 5 times while tumor sizes were monitored and recorded. After the last injection, mice were sacrificed, and plasma and tumor samples were collected for FACS analysis.

The anti-cancer activities of L-ATRA alone or combined with anti-PD1 were evaluated in the CT26 colorectal carcinoma mouse model. L-ATRA was injected every other day and anti-PD1 was given every three days, both by intravenous injection.

### Statistical Analysis

Results were presented as mean ± SD. Statistical analysis was carried out using either the Student’s t-test or one-way ANOVA in the excel software.

## Results

### Preparation and Characterization of L-ATRA

**Figure 1:**
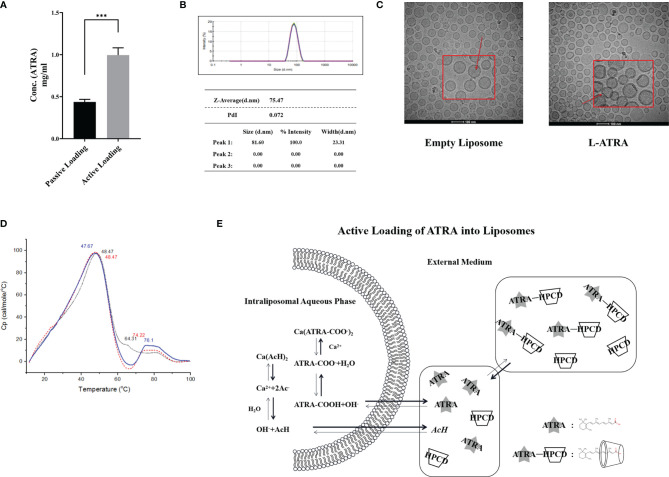
L-ATRA preparation and characterizations. **(A)** Concentrations of ATRA liposomes prepared using passive loading or active loading procedures; **(B)** Particle size distribution and PDI of L-ATRA; **(C)** Representative cryoEM images of empty liposomes (left) and L-ATRA (right); **(D)** The nano-DSC thermograms of L-ATRA; **(E)** Schematic drawing of the active loading mechanism for preparing L-ATRA. The p value for the symbol *** were < 0.001 (p=0.0005, student t-test).

ATRA is very poorly soluble in aqueous medium. Its solubility was about 0.21 μM at pH 7.3 and increased slightly at higher pH (**Supplementary Figure 1A**). It may be co-suspended in the lipid bilayer (i.e., passive loading). But using active loading mechanisms, the loading capacity would be much higher (**Figure 1A**). Extra-vesicular ATRA was driven into the liposomes by a calcium acetate gradient. The encapsulation efficiency (EE%) was higher than 90% when the loading D/L ratio (w/w) was about 0.06 (**Supplementary Figure 1C**). The actively loaded ATRA liposomes were named L-ATRA. The liposome sizes were about 75nm in diameter (**Figure 1B**) and did not change after loading. The zeta-potential was around -20mv. The drug release from L-ATRA were much slower than that from passively loaded liposomes (**Supplementary Figure 1E**).

The addition of HPCDs up to 10% (w/w) in the loading solution did not affect the liposome size (**Supplementary Figure 1B**). They were removed by dialysis after loading, so there was no detectable HPCD in the final L-ATRA formulation. We also examined the effect of incorporating HPCD inside the liposomes (**Supplementary Figure 1D**). However, the resulted loading efficiency was lower, suggesting that solubilizing ATRA inside the liposomes may not help the encapsulation stability. In addition, cryo-EM images implied that there were crystalline structures inside liposomes attached to the inner lipid layer (**Figure 1C**). NanoDSC scanning of L-ATRA revealed a new peak around 75°C in addition to the lipid phase transition peak around 48°C (**Figure 1D**). These data all suggested that ATRA was actively loaded inside liposomes where they precipitated as nano crystals. The L-ATRA preparation scheme was summarized in **Figure 1E**.

### L-ATRA Promoted Myeloid Cell Maturation and Differentiation Dose-Dependently

**Figure 2:**
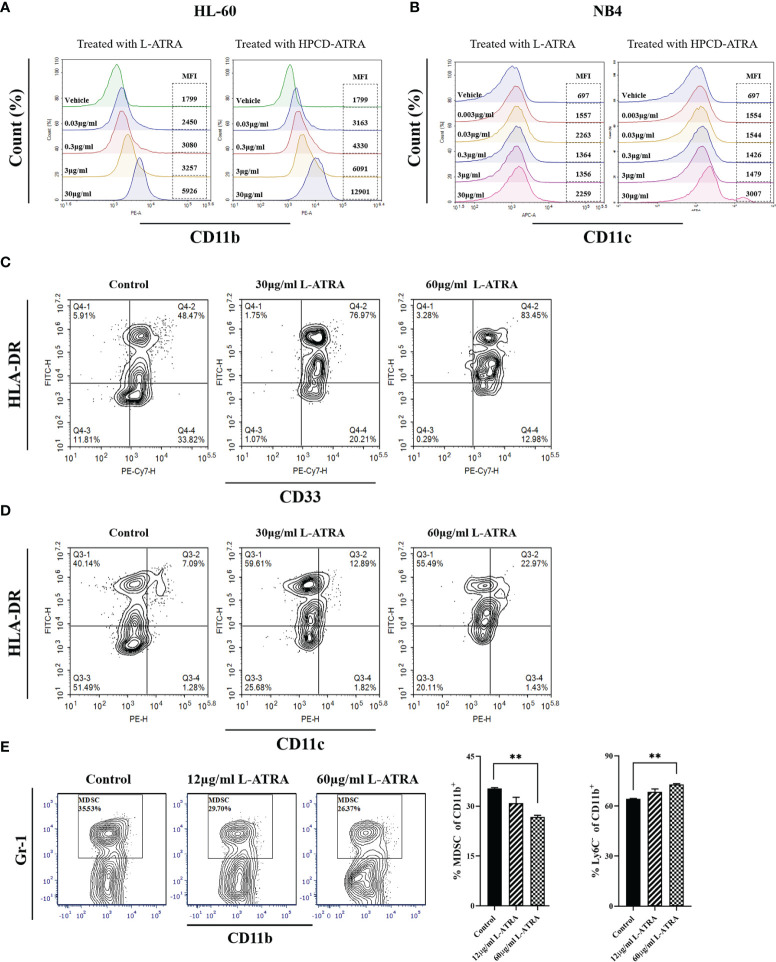
The *in vitro* L-ATRA dose effects on MDSCs. **(A, B)** The dose effects of L-ATRA on human leukemia cell line HL-60 cells and NB4 cells; **(C, D)** L-ATRA treatment of PBMCs isolated from cancer patient blood samples. **(E)** L-ATRA treatment of isolated CT-26 mouse tumor infiltrating myeloid cells. The percent of MDSCs in CD11b^+^ myeloid cells and percent of Ly6C^-^ cells in CD11b^+^ myeloid cells were also plotted as bar graphs on the right (**p < 0.01, one-way ANOVA analysis).

The myeloid leukemia cell lines HL-60 and NB4 were treated with L-ATRA as well as ATRA solubilized with HPCD. The cells responded to both treatments with similar dose dependency. Higher expressions of myeloid differentiation markers CD11b and CD11c were shown significantly at the ATRA dose of 30 µg/ml (**Figures 2A, B**).

**Figures 2C, D** plotted the phenotypical analysis of human peripheral myeloid cells from cancer patients before and after L-ATRA treatment. Due to the limited cell numbers available, we did not include the ATRA-HPCD control. The percent of HLA-DR^-^CD33^+^ cells (MDSCs) was greatly reduced, from 33.82% pre-treatment to 12.98% in the 60µg/ml dose group. Meanwhile, there were significantly more HLA-DR^+^CD11c^+^cells (DCs) after L-ATRA treatment (**Figure 2D**).

**Figure 2E** described tumor infiltrating CD11b^+^ cells isolated from CT-26 tumor bearing mice before and after L-ATRA treatment. L-ATRA was added into tumor cell conditioned culture medium containing 10ng/ml of mouse GM-CSF. The L-ATRA treatment reduced the number of MDSCs in a dose-dependent manner.

### L-ATRA Pharmacokinetics and Tissue Distribution

**Figure 3:**
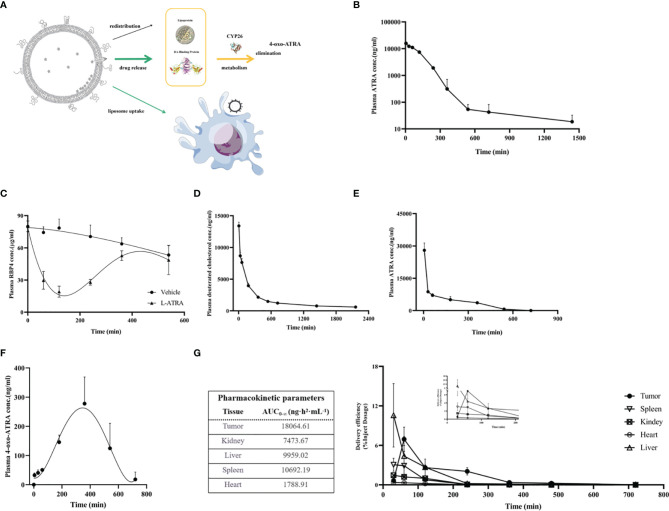
L-ATRA pharmacokinetics and tissue distributions after iv injection. **(A)** Schematic drawing of pharmacokinetics of L-ATRA *in vivo*; **(B)** Plasma ATRA concentration curves after L-ATRA i.v. injection in mice; **(C)** The measurements of RBP4 concentration changes after L-ATRA injection in mice; **(D–F)**. Plasma concentrations of **(D)** ATRA, **(E)** deuterated cholesterol, and **(F)** 4-oxo ATRA after L-ATRA i.v. injection in Beagle dogs; **(G)** ATRA concentrations in various tissues after L-ATRA i.v. injection in CT26 tumor bearing mice.

ATRA is an endogenous vitamin A that had been studies thoroughly concerning its distribution and clearance. The retinol binding proteins in the plasma are crucial for binding to and transport ATRA to various types of cells, where they were metabolized by CYP26s into 4-oxo-ATRA (**Figure 3A**). Based on such a mechanism, we examined the total ATRA plasma concentration, retinol binding protein 4 levels, as well as 4-oxo-ATRA concentrations after L-ATRA injection. In addition, we also labeled L-ATRA with trace amount of deuterated cholesterol and analyzed its pharmacokinetics. **Figures 3B, C** plotted the plasma concentrations of ATRA and RBP4 in mice. The clearance T_1/2_ was calculated to be 7.28 hours and the AUC was 411100 ng·h^2^·mL^-1^ (**Figure 3B**). Meanwhile, the plasma RBP4 level dropped quickly after L-ATRA injection and gradually return back after 6 hours (**Figure 3C**).

The PK study in beagle dogs showed similar trend. The liposomes were labeled with deuterated cholesterol in order to characterize the liposome PK properties. As shown in **Figure 3D, E**, liposomes remained in the circulation much longer than ATRA. Furthermore, the measurements of 4-oxo-ATRA in the plasma were the highest around 6-8 hours after injection (**Figure 3F**). All these data suggested that ATRA was gradually released from liposomes for more than 6-8 hours after injection.

The ATRA biodistribution in the various tissues in CT26 tumor bearing mice were analyzed and summarized in **Figure 3G**. Again, because released ATRA would be quickly metabolized, most of the measured ATRA concentration should be liposome encapsulated drug. There is clear trend of drug accumulation into the tumor tissue and the AUC was the highest among all the tissue types.

### The Anti-Tumor Effect of L-ATRA in Mouse Models

**Figure 4:**
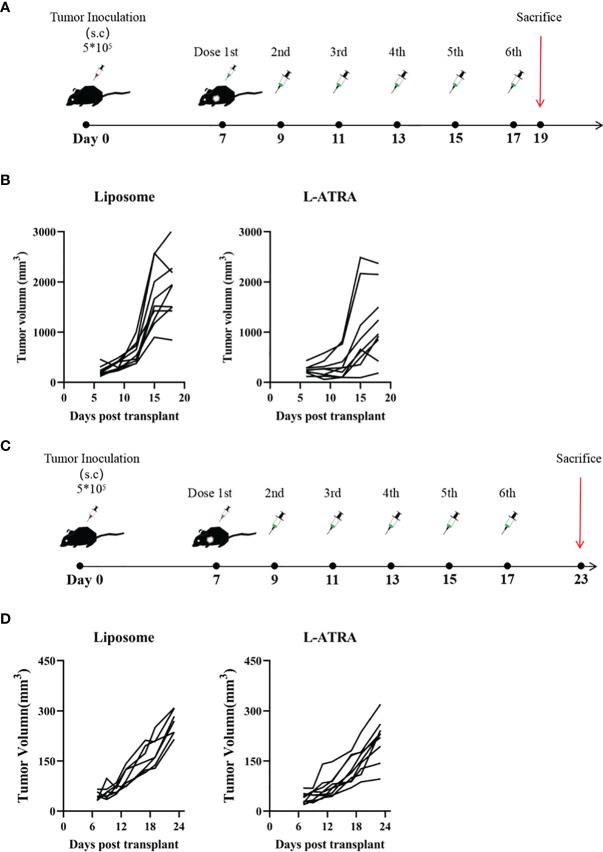
The tumor growth inhibition effect of L-ATRA in Syngeneic CT26 mouse colorectal cancer mice or A549 human lung cancer xenograft mice. **(A)** Syngeneic mice bearing CT26 mouse colorectal cancer were treated with L-ATRA every other day; **(B)** Tumor volume from each individual mice treated with either liposomes (left) or L-ATRA(right). **(C)** Nude mice bearing A549 human lung cancer received injections every other day for a total of 6 doses; **(D)** Tumor volume from each individual mice.

The anti-tumor effect of L-ATRA as an immunotherapy agent was examined in both immune competent mice and those with impaired immune systems. The syngeneic CT26 colorectal carcinoma model was established in immune competent Balb/C mice. After the tumors reached 100 mm^3^, they received either L-ATRA or blank liposomes *via* intravenous injection (**Figure 4A**). The tumor volume changes of each individual mouse in **Figure 4B** indicated that L-ATRA treated mice showed slower tumor growth. The two groups of data were analyzed using student’s t test (unpaired two-tailed). The effect of L-ATRA treatment was statistically significant (p =0.028).

For comparison, the A549 human lung carcinoma xenograft model was set up using nude mice with impaired immune system. After the same L-ATRA treatment (**Figure 4C**), the tumor growth was about the same as those in the empty liposome group (**Figure 4D**).

### The Effect of L-ATRA on Systemic Immune Cell Homeostasis

**Figure 5:**
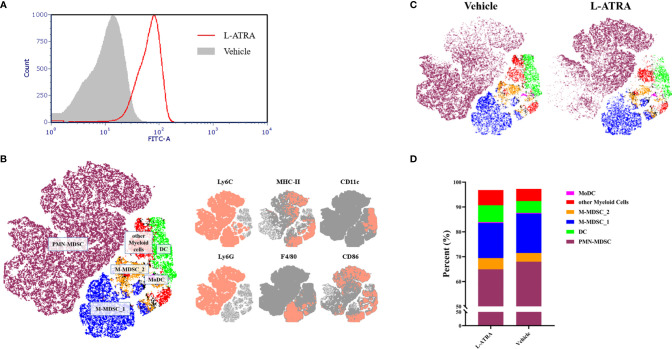
The analysis of PBMCs after L-ATRA treatments in syngeneic CT26 colon cancer mice. **(A)** The interactions between fluorescent labeled L-ATRA with primary PBMCs *in vitro*; **(B)** The merged t-distributed stochastic neighbor embedding (tSNE) plots of CD11b+ myeloid cells in PBMCs. Cells were profiled by flow cytometry and clustered for the identfication of various immune cell populations (left). The marker expression schemes were also shown (right); **(C)** Comparisons of the tSNE plots of CD11b^+^ myeloid cells in PBMCs with or without L-ATRA treatments; **(D)** The myeloid cell compositions in PBMCs with or without L-ATRA treatments.

The interaction of L-ATRA with purified CD11b+ myeloid cells from mouse peripheral blood mononuclear cells (PBMCs) were examined *in vitro*. L-ATRA was labeled with FITC-DSPE, and added into the myeloid cell culture and incubated for 4 hours. Majorities of the cells were labeled after the incubation (**Figure 5A**)

The pharmacodynamic effects after L-ATRA injections every two days for a total of 5 times were examined in syngeneic colorectal carcinoma (CT26) mouse models. The PBMCs were obtained and analyzed by FACS using a variety of myeloid markers. The data was further processed and presented as unsupervised t-SNE plots (**Figures 5B, C**). The t-SNE analysis collapsed complex multi-dimensional geometric relationships into a two-dimensional space plus a third dimension using a color-coded scheme. 6 myeloid cell clusters were identified, including the PMN-MDSCs, M-MDSCs and DCs (**Figure 5B**). The t-SNE plots with or without the L-ATRA treatment were compared side by side in **Figure 5C**. There were clear differences among the different clusters as well as within each clusters. There were many more MHC-II high expression PMN-MDSCs (purple colored) after the treatment. The composition of the different myeloid cell phenotypes were also affected (**Figure 5D**). The numbers of Ly6C high MHC-II positive M-MDSCs were reduced (14.08% vs. 15.84%) and the numbers of DCs were increased (6.78% v.s 4.73%).

### The Effect of L-ATRA on Tumor Infiltrating Immune Cells in the Tumor Microenvironment

**Figure 6:**
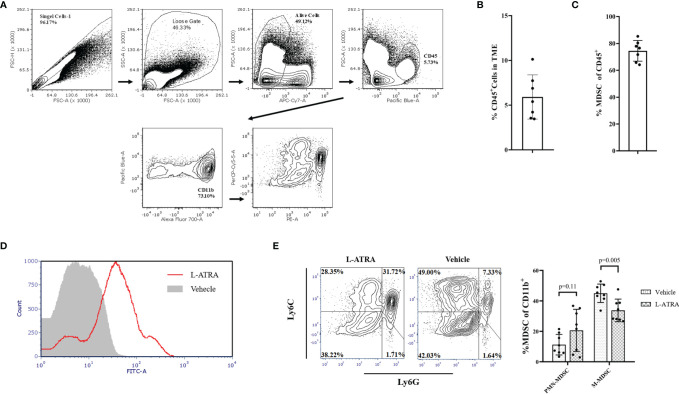
The effects of L-ATRA on modulating the tumor microenvironment. **(A)** Analysis scheme of the tumor infiltration myeloid cells; **(B)** The percent of CD45^+^ lymphocyte cells in tumor tissues; **(C)** The proportion of CD11b^+^ myeloid cellsin the tumor infiltration lymphocyte; **(D)** Binding of fluorescent labeled L-ATRA to CD11b^+^ tumor infiltrating myeloid cells *in vitro*; **(E)** FACS analysis of the tumor infiltrating myeloid cell after treatment with L-ATRA.

Tumor infiltrating immune cells were isolated from the tumor tissues by FACS sorting (**Figure 6A**). CD45^+^ lymphocyte cells accounted for about 5.9% of total cells in the tumor tissue and about 75% of them were CD11b^+^ myeloid cells (**Figures 6B, C**). L-ATRA was shown to be able to interact with these cells *in vitro* (**Figure 6D**). Based on these methods, we then analyzed the tumor infiltrating myeloid cells after 5 times of L-ATRA injections every other day. The myeloid cells were labeled with CD11b, Ly6C and Ly6G to identify the (CD11b^+^Ly6C^+^Ly6G^+^) PMN-MDSCs and (CD11b^+^Ly6C^+^Ly6G^-^) M-MDSCs. While there was a slight increase of PMN-MDSCs, the number of M-MDSCs in the tumor tissue was significantly reduced (30 ± 3.1% vs 23 ± 5.5%) (**Figure 6E**).

### The Anti-Tumor Effect of L-ATRA Combined With Anti-PD1

**Figure 7:**
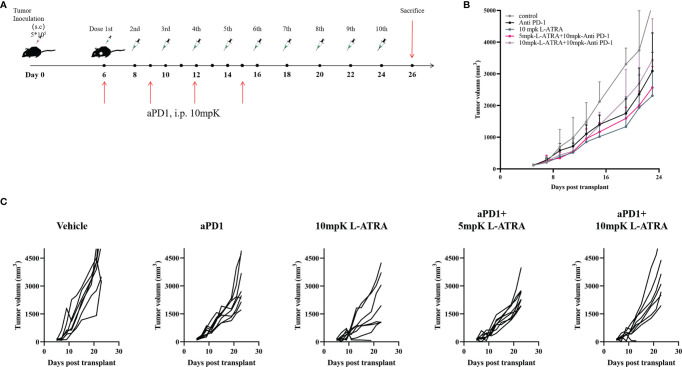
The tumor growth inhibition effects of L-ATRA combined with anti-PD-1 treatment. **(A)** Outline of the dose schedule; **(B)** Analysis of Tumor growth in different treatment groups; **(C)**. Individual tumor growth curves in different treatment groups.

The combined effect of L-ATRA and T cell checkpoint inhibitors were also examined. The syngeneic CT26 colorectal carcinoma tumor model mice were treated with L-ATRA at two dose levels, with or without concurant anti-PD-1 treatment (**Figure 7A**). As shown in **Figure 7B**. L-ATRA and anti-PD-1 combined treatment can consistently slow down tumor growth. The improvement is statistically significant (P<0.01). In comparison, anti-PD-1 by itself was only marginally useful, although the treatment regime was not optimized. The tumor volume from each individual mice in all these groups were shown in **Figure 7C**.

## Discussion

Oral administration of All-trans retinoic acid (ATRA) combined with chemotherapy is now the standard of care for treating Acute Promyelocytic Leukemia (APL) in adults (22). It was found to target the leukemia-causing fusion oncogene PML-RAR and promote myeloid cancer cell differentiation. The differentiation therapy mechanism has been well recognized and explored for the treatment of other cancer types (17). Wei et al. in their study suggested ATRA may also bind and degrade the peptidyl-prolyl cis/trans isomerase PIN1 in cancer cells (23). But there has been very limited clinical applications, partly because of the very poor solubility and bioavailability of ATRA (24). In our preliminary studies, we prepared L-ATRA and compared its dose effects for inhibiting cancer cell proliferation and modulating tumor-associated myeloid cells. We found that cancer cells became apoptotic when L-ATRA was dosed at 200μM and higher (data not shown). But the effective dose for eliminating MDSCs was around 40μM. Furthermore, we also found that L-ATRA was more effective in immune-competent mice than that in nude mice with impaired immune systems (**Figure 4**). Thus, we focused on the immune modulatory effects of L-ATRA in this study.

Many previous studies had investigated the central role of ATRA in orchestrating the differentiation, maturation and functionalities of myeloid lineage cells. Mirza and his colleagues showed that ATRA promoted differentiation of immature myeloid cells and improve tumor-specific immune response mediated by both CD4 and CD8 T cells (9). Bauer et al. reported that ATRA abrogated the accumulation of MDSC in tumor tissues in breast cancer models and increased the efficacy of anti-angiogenic therapy (25). Long et al. reported that treatment with ATRA largely eradicated M-MDSCs and diminished the suppressive capacity of PMN-MDSCs in sarcoma-bearing mice, which significantly improved the outcomes of CAR-T therapy (26). But there was a different study by Devalaraja et al. that proposed that tumor derived ATRA might promote intra-tumoral myeloid cell to differentiate into immune suppressive tumor associate macrophages (TAMs) (27). In this study, we used MDSCs collected from mouse tumor models and cancer patients and studied the dose effects of L-ATRA. These MDSCs were found to differentiate into mature DCs at about 12μg/ml ATRA concentration (**Figure 2C–E**). On the other hand, the endogenous ATRA concentration reported in the Devalaraja study was about 5-20nM. There may be a very distinct dose correlation of the PD effects. Therefore, we think it is very important to develop high concentration liposome formulations such as the L-ATRA to ensure the favorable immune modulation effects. Because of the poor solubility of ATRA, most earlier cell experiments studies applied ATRA in DMSO. L-ATRA can be dosed at concentrations as high as 5mg/ml, thus the pharmacodynamic effects on both circulating myeloid cells and tumor infiltrating myeloid cells were similar (**Figures 5**, **6**).

For the preparation of L-ATRA with improved apparent solubility and sufficient encapsulation stability, we developed the active loading mechanism as depicted in **Figure 1F**. ATRA is a small hydrophobic compound that’s highly sensitive to light, heat, and oxidation. What’s more, it has a very short biological half-life in humans (T_1/2 =_ 45min) and the endogenous ATRA concentrations in different tissues are tightly regulated (14). Besides, repeated oral ATRA dosing was found to cause a progressive reduction in plasma drug concentrations (28). There was an ATRA lipid formulation previous developed named ATRAGEN (29). But it was a lipid suspension containing ATRA incorporated by passive loading. The lipids acted mainly as solubilizing agents, so ATRA would quickly dissociated from the lipid carrier after injection and the drug plasma half-life was not improved (19, 30). The encapsulation stability of ATRAGEN and other suspension systems are also usually, so they require lyophilization during storage.

In our study, we adopted the calcium acetate active loading method (31) with some modifications. Specifically, we include HPCD in the loading buffer as the help to improve the trans-membrane drug concentration gradient. The ATRA concentration in 10% HPCD solution could reach 4-5mg/ml, while the liposomes maintained their integrity in the loading buffer. The encapsulated ATRA contributed to a new endothermal peak at ~ 75°C in DSC (**Figure 1E**). Based on the study of liposomes encapsulating doxorubicin by Wei et al. (32), the new co-operative melting peak may suggest the formation of drug containing nanocrystal inside liposomes which would also affected the drug release properties. Such crystalline structures were also observed in the cryoEM images shown as **Figure 1D**.

The actively loaded L-ATRA led to different drug release and pharmacokinetics behaviors compared to the passive loading control. **Figure 3A** probed the different aspects of L-ATRA drug release and distribution profiles after i.v. injection. While earlier studies on ATRAGEN showed ATRA dissociation from the lipid carriers soon after the injection, we found that there was a sustained release phase of L-ATRA in circulation (**Figure 3B**). Unlike doxorubicin encapsulated liposomes in which drug release in circulation is very slow, L-ATRA released drug much faster because ATRA has very high plasma protein binding tendency and fast metabolizing rates. Drug release led to a decrease of RBP4 levels (**Figure 3C**) and increase of 4-oxo-ATRA concentrations around 6-8 hours after injection (**Figure 3F**). The differences between the ATRA PK curve and deuterated cholesterol PK curve also supported the sustained release mechanism (**Figures 3D, E**). More importantly, the prolonged half-life of L-ATRA also resulted in liposome extravasate and accumulate in the tumor tissue due to the EPR effect. The ATRA AUC in the tumor tissues was higher than all other tissues including the liver and spleen (**Figure 3G**).

The unique pharmacokinetic behavior of L-ATRA resulted in its PD effects also in two folds. The sustained release of ATRA in circulation contributed to the rebuilding of the systemic immune homeostasis, and the drug accumulated in tumor tissue helped to modify the TME. Numerous studies had indicated that higher numbers of MDSCs in the peripheral may cause profound systemic immune suppression and interfere with various treatment outcomes in cancer patients (33, 34). Meyer et al. analyzed MDSCs in peripheral blood of advanced melanoma patients receiving ipilimumab and observed a significantly correlations between frequencies of circulating MDSCs and clinical responses (7). Reduction of MDSCs in peripheral blood was found to reverse the suppression of T cells and improve IFN-γ production (35). Oral dosing of ATRA had been used in combination with other agents to modulate MDSCs. Chiappori et al. applied ATRA to deplete MDSCs and aid immune responses in a p53-DC vaccine. Long et al. showed ATRA could enhance the efficacy of CAR-T therapies against solid tumors (26). The L-ATRA treatment in our study had similar effects (**Figure 5B**). The t-SNE analysis of myeloid cells in PBMCs revealed higher expressions of MHC-II in various cells, which may help to restore proliferation and activities of T cells especially antigen-specific CD4+ T cells (36). There were also a reduction of M-MDSCs and increased numbers of DCs in the circulation which should be valuable for improving CD8^+^ T cell activities in cancer patients (7, 12).

The other important aspect of the L-ATRA anti-tumor activities should be its modulation effects on the TME. The TME is considered as one of the most important factors in cancer treatment. The presence of various immune suppressive cells including Tregs, TAMs, and MDSCs were identified in the TME (37). The M-MDSCs were shown to suppress T-cell responses by producing high levels of NO and expressing immunosuppressive cytokines (38). They were recruited into tumor tissues in significant numbers and their presence promoted tumor progression and metastasis. We analyzed the CT26 tumor tissues and found significant amounts (~75%) of the tumor infiltrating lymphocytes were in fact MDSCs (**Figures 5B, C**). L-ATRA may interact with the MDSCs and release ATRA for extended period of time. Thus, the tumor infiltrating myeloid cells were significantly impacted, especially the M-MDSCs were greatly reduced (**Figure 5E**).

The use of nano drug delivery systems especially liposomes had achieved great successes taking advantages of the EPR effect. But most of them were developed targeting cancer cells, but there have been surging interests in the possibilities of modulating the TME (39). In order to isolate the immunomodulatory effect of L-ATRA and evaluate its contribution to cancer treatment, we examined the efficacy of L-ATRA as a single agent treatment. The PD effects of sustained ATRA release in circulation as well as in the TME were shown in both **Figures 5**, **6**. These changes reflect different mechanisms but both are required for restoring immune responses against cancer cells. In fact, cancer cell derived exosomes were found to interact with MDSCs in promoting tumor progression and metastasis (40). L-ATRAs are similar in size as exosomes, but ATRA can reverse the immune suppressive nature and correct MDSCs. Furthermore, since recent studies reveals that anti-PD-1 resistance may also be due to MDSCs (41), it would be highly desirable to combine MDSC targeted L-ATRA with T cell based check point inhibitors. The resulted efficacies looked promising (**Figure 7**). Further studies are needed in order to understand the interaction between the two immune modulation effects and optimize the combination regime for breaking tolerance and escape resistance.

In summary, we developed a new formulation of ATRA that has sustained drug release properties. It was shown to be able to modulate myeloid cell functions both systemically and within the TME. Since ATRA is a known drug with defined metabolic and safety mechanisms, L-ATRA may be a promising new IO agent for cancer threatment.

## Data Availability Statement

The raw data supporting the conclusions of this article will be made available by the authors, without undue reservation.

## Ethics Statement

Ethical review and approval was not required for the study on human participants in accordance with the local legislation and institutional requirements. The patients/participants provided their written informed consent to participate in this study. The animal study was reviewed and approved by Shanghai Jiao Tong University Animal Care and Use Committee.

## Author Contributions

AZ and YX contributed to conception and design of the study and wrote the manuscript. AZ contributed to organized the database and performed the statistical analysis. FX, SS, SL, and JL wrote sections of the manuscript. All authors contributed to manuscript revision, read, and approved the submitted version.

## Funding

We acknowledge the grant support from the National Natural Science Foundation of China (NSFC) No. 31571019 and Yunnan Department of Science and Technology #202105AE160010.

## Conflict of Interest

The authors declare that the research was conducted in the absence of any commercial or financial relationships that could be construed as a potential conflict of interest.

## Publisher’s Note

All claims expressed in this article are solely those of the authors and do not necessarily represent those of their affiliated organizations, or those of the publisher, the editors and the reviewers. Any product that may be evaluated in this article, or claim that may be made by its manufacturer, is not guaranteed or endorsed by the publisher.
