# Supplementary material for: Sustained Drug Release From Liposomes for the Remodeling of Systemic Immune Homeostasis and the Tumor Microenvironment
Source: Front Immunol. 2022 Apr 12;13:829391. doi: 10.3389/fimmu.2022.829391 (PMC9039229; doi:10.3389/fimmu.2022.829391)
Supplement: Supplementary file 1 [file DataSheet_1.pdf]

Supplementary Data

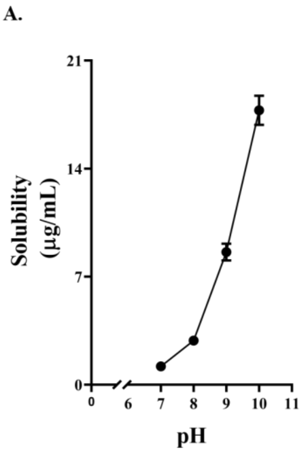

B.

The effect of additional HPCD in the drug loading buffer onLiposome size and PDI		
**Liposome**	**Before Loading**	**After Loading**
**Size**	75.74	75.47
**PDI**	0.052	0.072

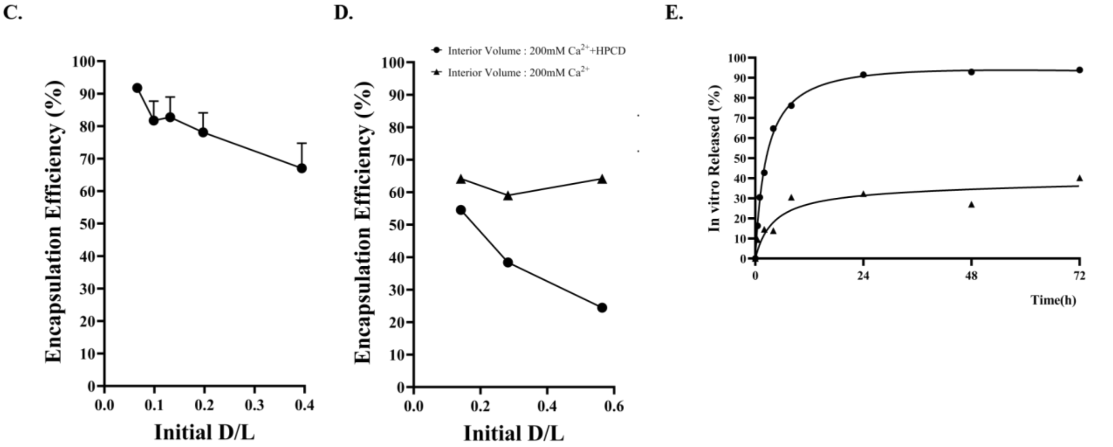

Supplement Figure 1. (A) the solubility of ATRA under different pH conditions; (B) encapsulation efficiency of liposome with different interior volume; (C) Encapsulation efficiencies of L-ATRA with different initial drug to lipid ratios; (D) The effects of HPCD addition in the drug loading buffer on ATRA encapsulation efficiencies; (E) ATRA releasing from L-ATRA(triangle)and passively loaded liposomes (dot) in medium containing 20% of serum.

REAGENT or RESOURCE	SOURCE	IDENTIFIER
Antibodies		
Alexa Fluor 700 anti-mouse CD11b	Biolegend	101222
PE/cy7 anti-mouse F4/80	Biolegend	123114
Pacific Blue anti-mouse CD45	Biolegend	103126
Brilliant Violet 510 anti-mouse CD86	Biolegend	105039
PE anti-mouse Ly6G	Biolegend	127607
PerCP/Cy5.5 anti-mouse Ly-6C	Biolegend	128011
Alexa Fluor 488 anti-mouse I-A/I-E	Biolegend	115008
TruStain fcX(anti-mouse CD16/32)	Biolegend	101320
Fixable viability dye efluor 780	eBioscience	88-8824-00
FITC anti-Human HLA-DR	Biolegend	980402
PE-Cy7 anti-Human CD33	Biolegend	366617
PE anti-Human CD11c	Biolegend	337224

Supplement Figure 2. List of antibodies used in this study.

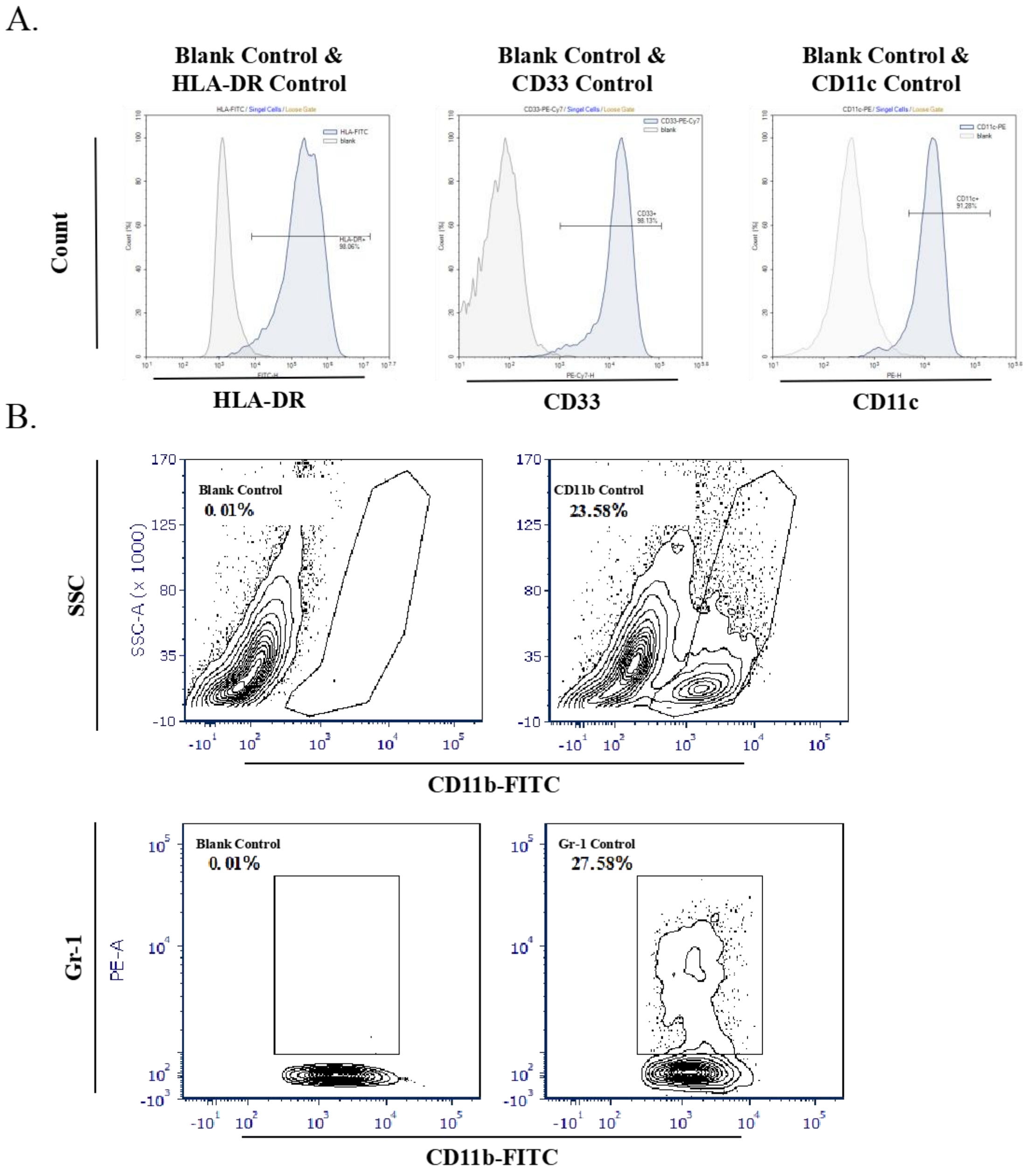

Supplement Figure 3. (A). Single parameter histograms of positively labeled cells for determing the HLA-DR, CD33, and CD11c gating threshold; (B) CD11b and Gr-1 single label contour plots and the gating strategies.
